# Synthesis of nanosize zinc oxide through aqueous sol–gel route in polyol medium

**DOI:** 10.1186/s13065-022-00900-3

**Published:** 2022-11-24

**Authors:** Samreen Zahra, Hamim Bukhari, Saboora Qaisar, Asma Sheikh, Athar Amin

**Affiliations:** 1 Mineral Processing Research Centre, PCSIR Laboratories Complex, Ferozepur Road, Lahore, 54600 Pakistan; 2 Department of Chemistry, Post Graduate Islamia College, Cooper Road, Lahore, 54000 Pakistan; 3 Food and Biotechnology Research Centre, PCSIR Laboratories Complex, Ferozepur Road, Lahore, 54600 Pakistan

**Keywords:** Zinc oxide, Sol–gel process, Polyol system, Nanoparticles, Crystal structure, Surface morphology

## Abstract

**Background:**

This study is aimed to synthesize nanosize zinc oxide by acid catalyzed sol–gel process using zinc nitrate hexahydrate as precursor, aqueous isopropanol as solvent and glycerin for making polyol system. The polyol mediated procedure was employed in combination with calcination induced synthesis of nanoparticles of numerous sizes obtained with the variation in calcination temperature from 500 to 900 ℃. The crystal structure of the prepared samples was characterized by X-ray diffraction analysis (XRD). Infrared spectroscopy (IR) was used to identify the surface hydroxyl groups. Thermal stability was confirmed by differential scanning calorimetry-thermogravimetric analysis (DSC-TGA) whereas field emission scanning electron microscopy (FESEM) was used to study the surface morphology of nanoparticles.

**Results:**

Results revealed the formation of hexagonal wurtzite structure of irregular shaped nanoparticles having size ranging from 50–100 nm. However, the particles combined to form agglomerates of 200–400 nm with the rise in calcination temperature.

**Conclusions:**

These results indicate that nanosize zinc oxide can be synthesized successfully by a simple process comprising of glycerin as a low-cost, non-toxic and eco-friendly polyol followed by calcination at ambient temperatures.

## Introduction

Zinc oxide (ZnO) has been found to be of great importance since it exhibits such semiconducting, pyroelectric, piezoelectric, optoelectronic and catalytic properties that make it a multi-functional material for use in biosensors, light emitting diodes, field effect transistors, ferromagnetic materials for spintronics solar cells, photocatalysis, antibacterial and antioxidants. It possesses a wide band gap of 3.37 eV with high binding energy i.e. 60 meV. Nanoscale ZnO possesses improved properties as compared to its bulk counterpart and therefore can be used in numerous products including rubber, electrophotography, photoprinting, capacitors, protective coatings, anti-microbial, and conductive thin-films in LCDs, blue laser diodes medicines, cosmetics and food items [1–3]. Gunalan et al. found zinc oxide nanoparticles quite active against various bacterial and fungal pathogens and proposed that these nanoparticles can be efficiently employed in agriculture and food safety [4].

As reported previously, a variety of conventional methods have been employed for the synthesis of zinc oxide nanoparticles like sol–gel method, anodization, co-precipitation, ultrasound, chemical vapor deposition (CVD) and mechanochemical-thermal synthesis [5]. Yahya and co-workers prepared zinc oxide nanoparticles by precipitation and self-combustion method for their potential use as a solar cell [6]. However, synthesis of zinc oxide by sol–gel method has been found to be simple and capable of producing particles with smaller size having larger specific surface area and high purity as compared to the other methods. Sol–gel synthesis of ZnO has been reported by many of the previous scientists [2, 7–9]. Recently, Vishwakarma et al. obtained ultra-fine zinc oxide particles of average particle size 58.3 nm via sol–gel process [10]. More recently, Somoghi et al. synthesized zinc oxide coatings modified with different silane coupling agents i.e. octyltriethoxysilane, octadecyltriethoxysilane and 3-glycidyloxypropyl trimethoxysilane using sol–gel method and concluded that these coatings can be highly suitable for industrial applications [11]. During the last decade, some researchers have adopted procedures mediated by polyalcohols (diethylene glocol, triethylene glycol, polyvinyl alcohol) for the synthesis of zinc oxide nanorods, nanoclips and nanospheres [12–17].

The current study has therefore been carried out to synthesize zinc oxide particles through polyol mediated sol–gel method followed by calcination induced process at various temperatures i.e. 500 ℃, 700 ℃ and 900 ℃. The phase composition, purity, thermal stability and surface morphology of the prepared samples were studied using X-ray diffraction technique, infrared spectroscopy, differential thermal analysis and field emission scanning electron microscopy.

## Experimental

### Reagents

Zinc nitrate hexahydrate (Sigma–Aldrich; 99%), isopropyl alcohol (WINLAB; 99%), glycerin (BDH; 99%) and nitric acid (Merck; 65%).

### Synthesis of zinc oxide nanoparticles

**Fig. 1:**
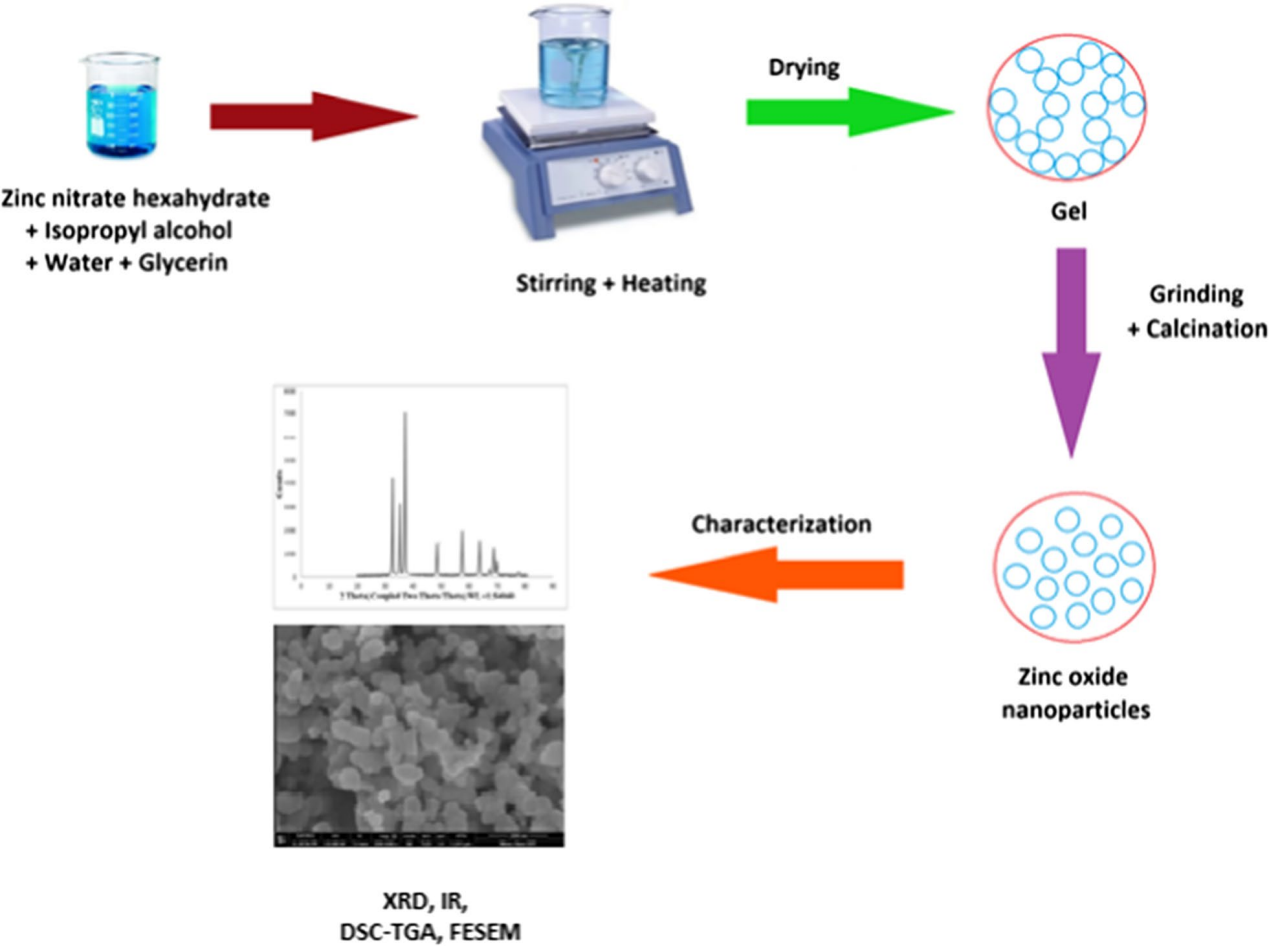
Graphical representation of synthesis of zinc oxide nanoparticles

Zinc oxide nanoparticles were synthesized through aqueous sol–gel route in acidic medium. 14.64 g of zinc nitrate hexahydrate were added to a mixture of solvents i.e. isopropyl alcohol and water in 1:4 ratio. 10 mL of glycerin were added and pH of the aqueous solution was maintained at 1 using nitric acid. The mixture was stirred constantly for two hours at 70 ℃. The sol formed was also dried at 70 ℃ for 24 h. The gel thus obtained was ground and was divided into three portions that were calcined for two hours at 500 ℃, 700 ℃ and 900 ℃ and were labeled as ZNA-1, ZNA-2 and ZNA-3 respectively. The graphical representation of the process is shown in Fig. 1.

### Characterization

The crystal structure of all the prepared zinc oxide samples was identified by Bruker D2-Phaser X-ray diffractometer by monochromatised CuK_α_1 radiation at a wavelength of 1.54060 Å. IR spectroscopy was performed with Thermo Nicolet IR 200 (USA). DSC-TGA was carried out using differential scanning calorimeter Universal V4.5A, TA instruments USA. Surface morphology was studied using field emission scanning electron microscope FEI Nova 450 NanoSEM.

## Results and discussion

Zinc oxide nanoparticles were prepared via polyol mediated solgel process and were calcined at 500 ℃, 700 ℃ and 900 ℃. The synthesis was carried out in acidic medium using aqueous isopropanol as solvent and glycerin as a polyol. A previous investigation revealed that surface morphology and other physical characteristics of metal oxide nanoparticles are highly dependent on the nature of solvents used. Solvent like isopropanol plays a vital role in enhancing the physical properties of nanoparticles thereby improving their catalytic properties manifolds as compared to their bulk counterparts [18]. Another observation about metal oxide nanoparticles obtained through aqueous systems is that they are commonly formed as hard agglomerates that results in reduction of their surface area and hence affects their performance in various applications. The polyol medium, on the other hand, has proved to be a suitable alternative for inhibiting agglomeration since the polyol acts as both solvent as well as stabilizing agent. Moreover, use of high boiling polyol has been found to be more appropriate [19]. In this study, glycerin was therefore employed for controlling the nucleation and growth of zinc oxide nanoparticles. The prepared samples were characterized and results obtained are as follows:

### X-ray diffraction analysis

**Fig. 2:**
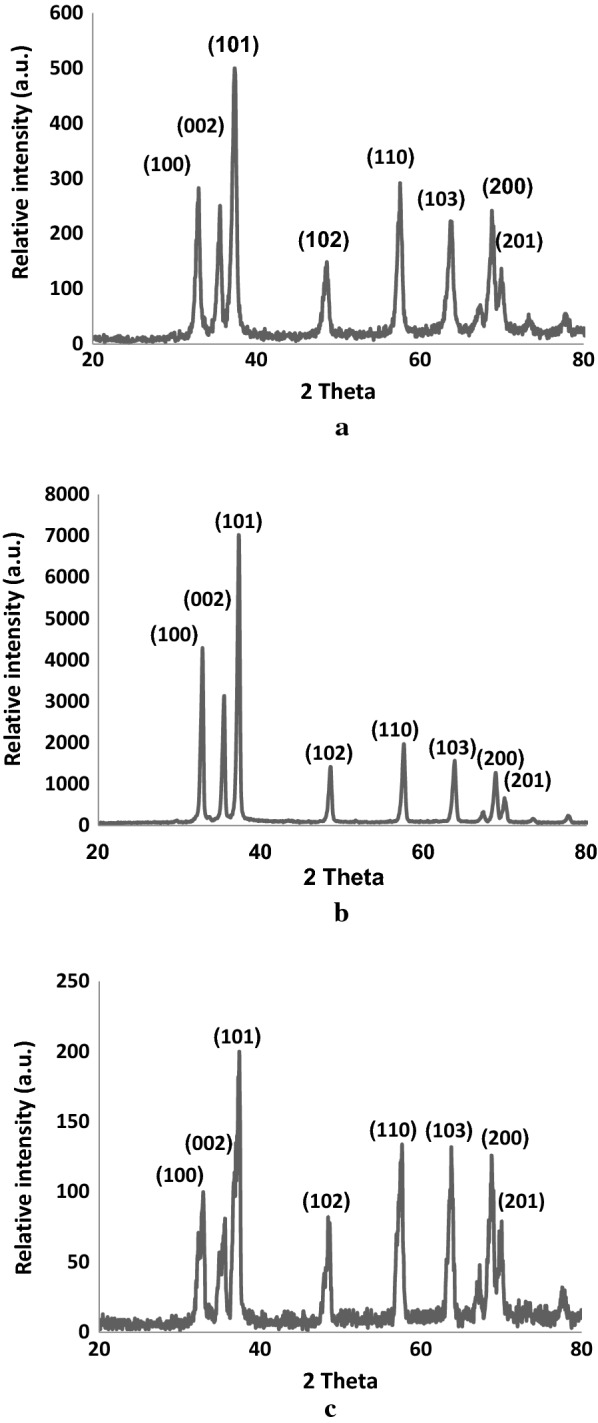
**a** X-ray diffraction pattern of ZNA-1. **b** X-ray diffraction pattern of ZNA-2. **c** X-ray diffraction pattern of ZNA-3

X-ray diffraction analysis of the synthesized ZNA samples was conducted to identify their crystalline structure and phase purity and diffractograms are illustrated in Figs. 2a, b and c. As observed in figures, the diffraction patterns for ZNA-1, ZNA-2 and ZNA-3 calcined at 500 ℃, 700 ℃ and 900 ℃ respectively show various peaks corresponding to the hexagonal wurtzite phase of zinc oxide. The major peaks appear at 2θ values 31.93° and 36.33° corresponding to (100) and (101) planes while the other peaks confirming the presence of wurtzite structure can be seen at 2θ values 34.56°, 47.61°, 56.56°, 62.73°, 67.79° and 69.95° consistent with (002), (102), (110), (103), (200) and (201) planes (JCPDS Card no: 36–1451). These results are in close agreement with the observations of previous scientists [1, 2, 15, 20–23].

No other peaks are observed in the patterns which indicate the absence of impurities. Hence, XRD results reveal that similar diffraction peaks appear in case of all the three ZNA samples with no significant variation in intensities except ZNA-2 in which the major diffraction peaks appear to be relatively sharp as compared to ZNA-1 and ZNA-3. This observation can be attributed to lattice variation with the rising calcination temperature owing to the presence of dangling bonds on the surface of zinc oxide nanoparticles. Kayani et al. observed a decrease from 300 to 500 ℃ followed by an increase in lattice constants with further rise in calcination temperature [24].

### Infrared spectroscopy

**Fig. 3:**
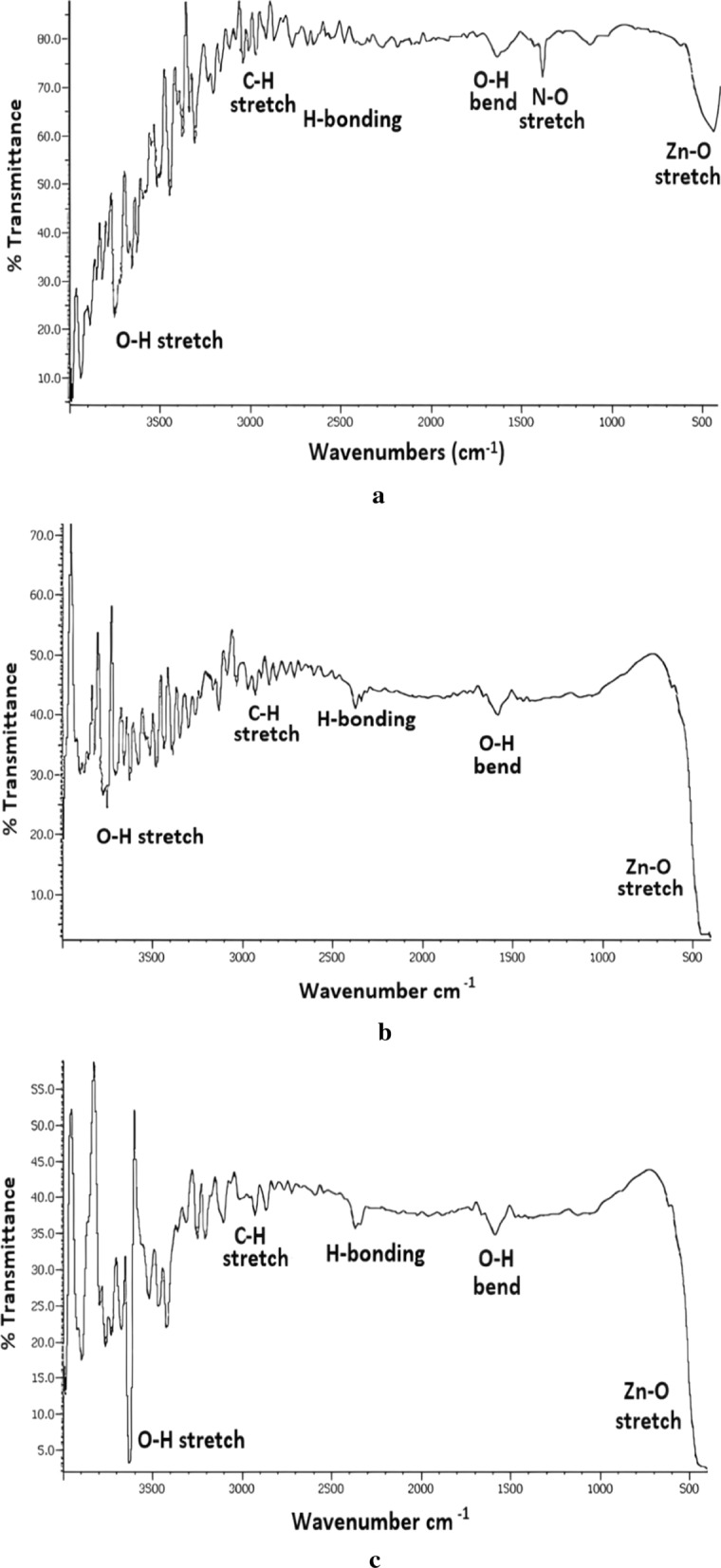
**a** IR spectrum of ZNA-1. **b** IR spectrum of ZNA-2. **c** IR spectrum of ZNA-3

The IR spectra of ZNA samples recorded in the frequency range of 4000–400 cm^−1^ are demonstrated in Figs. 3a, b and c. The spectra depict similar absorption bands with slight variation in intensities among all the samples. In the region between 3800 and 3000 cm^−1^ several absorption bands combined to form a broad band can be seen. This region corresponds to the combination of various stretching vibrations of hydroxyl groups of surface adsorbed water molecules and hydrogen bonded hydroxyl groups [25, 26]. However, it can be seen that absorption bands become relatively distinct with the rise in calcination temperature probably due to particle growth.

The bands of minor intensities between 3000 and 2800 cm^−1^ can be ascribed to the stretching vibrations of alkyl groups of organic impurities entangled in the nanocrystals during their synthesis [27]. Many less intense absorption bands in the region 2800–2200 cm^−1^ appear due to strong hydrogen bonding between water molecules. The absorption bands around 1600 cm^−1^ are attributed to the bending vibrational modes of hydroxyl groups of water molecules [28–30]. Another minor absorption band at 1384.72 cm^−1^ associated with asymmetric stretching vibrations of nitrate group can be observed in case of ZNA-1, however, no such bands appeared for ZNA-2 and ZNA-3 [31].

The prominent broad band below 1000 cm^−1^ with high intensity in case of ZNA-2 and ZNA-3 and relatively less intensity for ZNA-1, signifies the presence of Zn–O bonds of zinc oxide since metal oxides generally exhibit absorption bands in fingerprint region below 1000 cm^−1^ [32]. Recently, Jayachandran et al. obtained multiple sharp bands at 491 cm^−1^ and 435 cm^−1^ attributed to the presence of Zn–O bonds [33]. Mahamuni and co-workers also observed bands corresponding to the stretching vibrations of Zn–O around 415–480 cm^−1^. According to them slight variations in wavenumbers and frequencies are consistent with the variety of particle sizes of metal oxides [15].

### Thermal analysis

**Fig. 4:**
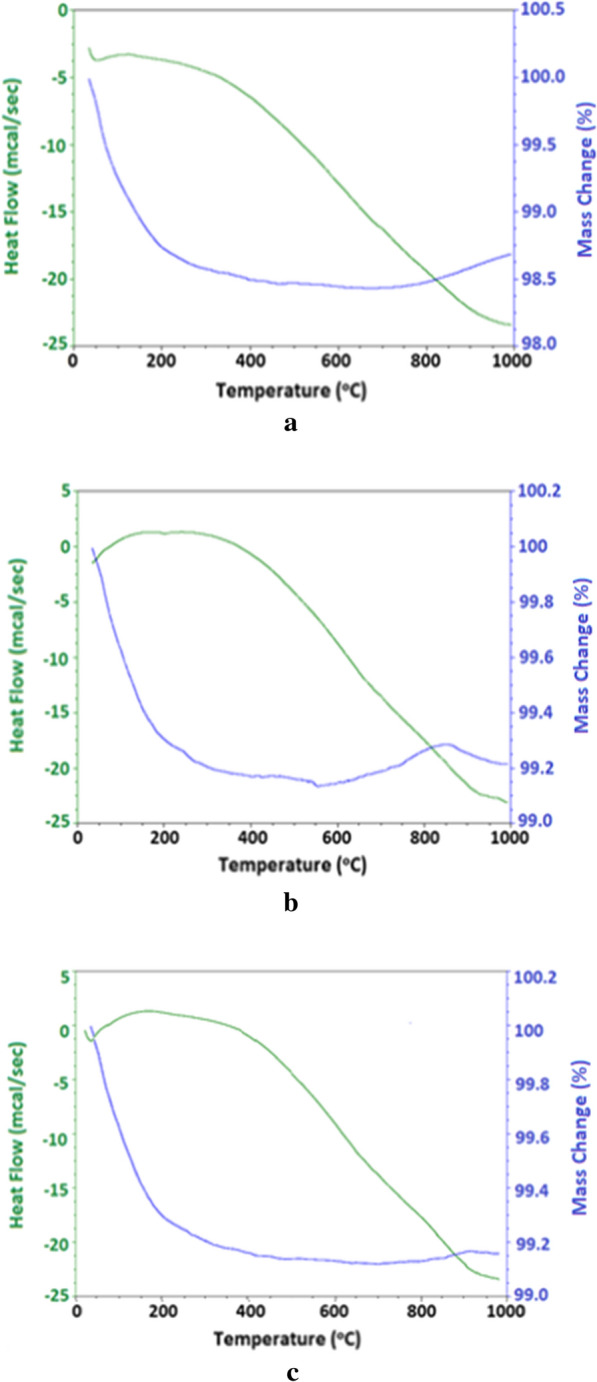
**a** DSC-TGA curves of ZNA-1. **b** DSC-TGA curves of ZNA-2. **c** DSC-TGA curves of ZNA-3

Thermal behavior of the prepared zinc oxide samples ZNA-1, ZNA-2 and ZNA-3 was investigated by DSC-TGA and curves are shown in Figs. 4a, b and c respectively. The TGA curve presented in Fig. 4a offers a two-step loss below 700 ℃ in case of ZNA-1. In the initial step, an abrupt weight loss of 1.26% with a corresponding endothermic peak shown in DSC curve till 200 ℃ is ascribed to the evaporation of adsorbed moisture [34]. In another step in the temperature range of 200–690 ℃, a gradual weight loss of 0.31% resulted due to the removal of organic residues entrapped between the crystals during their synthesis [15]. In addition, a negligible weight gain of 0.03% noticed from 690 to 1000 ℃ can be attributed to the heat transfer between sample and crucible. Both these stages are accompanied with an endothermic peak that continues from 200 to 1000 ℃ with a gradual but continuous change in heat absorbed by the sample.

Figure 4b illustrates a net weight loss of 0.78% for ZNA-2 with four stages of trivial changes in weight i.e. three stages of weight loss analogous to ZNA-1 while an additional stage comprising of a weight gain of 0.14% from 550 to 870 ℃ due to organic residues entrapped by the mesopores present between the crystallites. However, the resulting DSC curve appears to be similar to that of ZNA-1. ZNA-3 (Fig. 4c) also showed a weight loss of 0.82% ascribed to physically adsorbed moisture and organic residues with a corresponding endothermic peak exhibited in DSC curve like ZNA-1. Among previous scientists Khan et al. observed 48.14, 48.30, 51.34 and 55.55% residues among various zinc oxide samples indicating 51.86, 51.70, 48.66 and 44.45% weight loss respectively at 450 °C [8]. However, thermal changes observed among the synthesized ZNA samples indicate complete transformation of the precursor to zinc oxide and hence, confirm their purity.

### Field emission scanning electron microscopy

**Fig. 5:**
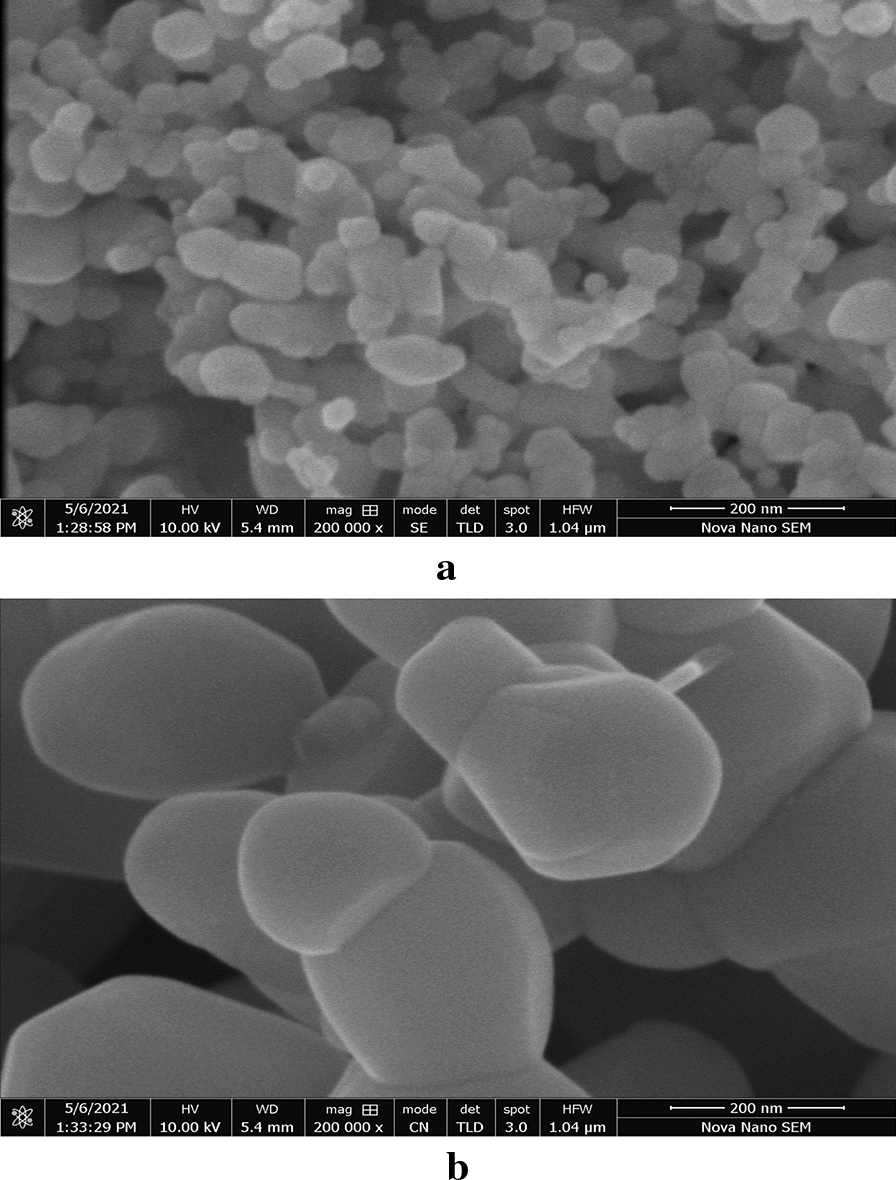
**a** FESEM micrograph of ZNA-1. **b** FESEM micrograph of ZNA-3

Surface morphology of ZNA-1 and ZNA-3 was studied by field emission scanning electron microscopy. The images recorded at 200,000 × magnification are presented in Figs. 5a and b respectively. Figure 5a shows uniform distribution of nanoparticles having irregular shapes and different sizes ranging from 50 to 100 nm with a slight tendency to agglomerate. The micrograph for ZNA-3 (Fig. 5b) also illustrates irregular morphology however, the particles appear to be larger in size as compared to ZNA-1 due to the particle growth at higher calcination temperatures [35].

In fact, increase in calcination temperature results in a rapid growth in nucleation rate of the particles owing to the super-saturation of products making crystal core in a very short period of time causing nuclear aggregation with the continuous rise in temperature. Parra et al. observed increase in crystallite size from 28 to 44 nm with the rise in calcination temperature from 200 to 500 ℃ [36]. The agglomeration among nanoparticles also occurs due to their tendency to lower the high surface energy during their growth [37]. Actually the extent of particle aggregation is the key factor that affects the surface morphology and structure of the final product. Comparison of morphology of zinc oxide nanoparticles prepared by different routes is given in Table 1.

**Table 1:** Comparison of morphology of zinc oxide nanoparticles prepared by different routes

Synthesis method	Shape	Size	References
Wet chemical synthesis	Horizontal/Agglomerated	52.24 nm	Jayachandran et al*.* [33]
Microwave–assisted	Nanoflakes/Nanoplates	60–200 nm	Yalcin et al*.* [38]
Green synthesis	Hexagonal	40–45 nm	Alamdari et al*.* [39]
Co–precipitation	Spherical/Aggregated	80 nm	Mahmood et al*.* [40]
Precipitation	Spherical	20–40 nm	Ghorbani et al*.* [41]

## Conclusion

Present study leads to the conclusion that zinc oxide nanoparticles have been synthesized successfully through sol–gel process using aqueous isopropanol as solvent and glycerin as a low-cost, non-toxic and eco-friendly polyol. A distinct combination of polyol mediated procedure and calcination induced process effectively controlled agglomeration leading to the formation of nanoparticles of different sizes between 50 and 100 nm. However, the particles combined to form agglomerates of 200–400 nm size obtained with the rise in calcination temperature from 500 to 900 ℃. X-ray diffraction analysis confirmed the formation of hexagonal wurtzite structure of zinc oxide. Hence, the study reveals a simple process for the synthesis of zinc oxide nanoparticles.

## Data Availability

All data generated or analyzed during this study are included in the manuscript.
